# Nociceptive Sensory Fibers Drive Interleukin-23 Production in a Murine Model of Psoriasis *via* Calcitonin Gene-Related Peptide

**DOI:** 10.3389/fimmu.2021.743675

**Published:** 2021-10-22

**Authors:** Xuan Zhang, Jiali Cao, Siqi Zhao, Xutong Yang, Jie Dong, Yaqi Tan, Teng Yu, Yanling He

**Affiliations:** ^1^ Department of Dermatology, Beijing Chao-Yang Hospital, Capital Medical University, Beijing, China; ^2^ National Clinical Research Center for Skin and Immune Diseases, Branch in Beijing Chaoyang Hospital, Beijing, China

**Keywords:** psoriasis, neuroimmunity, calcitonin gene-related peptide, nociceptive sensory neurons, type17 immune response

## Abstract

Neuroimmunity is involved in the pathogenesis of psoriasis, but the mechanism underlying the interaction between the nervous system and the interleukin (IL)-23/IL-17 immune axis is yet unclear. This study reveals the essential role of the sensory neuron-derived calcitonin gene-related peptide (CGRP) in imiquimod (IMQ)-induced expression of IL-23. First, we show that the increased nociceptive behavior was consistent with the development of psoriasiform dermatitis, which requires intact sensory innervation. Systemic ultrapotent Transient receptor potential vanilloid 1 (TRPV1) agonist (resiniferatoxin, RTX) treatment-induced sensory denervation resulted in a significant decrease in IL-23 expression in this model, while the recombinant IL-23 treatment induced IL-17A expression was intact after RTX treatment. In addition, IMQ exposure induced a transient increase in CGRP expression in the dorsal root ganglion. The neuron-derived CGRP expression was completely abolished by sensory denervation, thereby downregulating IL-23 expression, which could be reversed through the introduction of CGRP into the denervated dorsal skin. Our results suggest that nociceptive sensory neurons may drive the production of IL-23, resulting in IL-17A production from γδ T cells *via* the neuropeptide CGRP in the pathology of psoriasis.

## Introduction

Psoriasis is a chronic, recurrent skin inflammatory condition that affects 2-3% of the world’s population. The disease is clinically characterized with the presence of scaly skin plaques, arthritis, and an inflammatory cell-rich cutaneous microenvironment (1). It is widely accepted that the interleukin (IL)-23/IL-17 axis plays a vital role in psoriasis development (2), as antibodies targeting IL-12/23 and IL-17 have revolutionized psoriasis treatment (3). Environmental and psychological stressors can exacerbate the symptoms of psoriasis and provoke flares. Intact innervation is essential to maintain the psoriatic lesion, and the central or peripheral nerve damage results in spontaneous remission of psoriatic skin lesions (4). Thus, the pathophysiology of psoriasis is multifactorial and comprises neurological and immunological factors. The skin is a highly sensitive organ, abundantly innervated by primary sensory nerve endings that carry tactile information to the brain (5). Transient receptor potential vanilloid 1/ankyrin 1 (TRPV1)/(TRPA1)-positive sensory fibers have been well characterized in the skin, and are known to respond to various stimuli, including capsaicin, noxious temperature, and other stimuli. The abnormal activation of TRPV1/TRPA1 channels can result in pruritus and hyperalgesia, and accounts for the clinical phenomena experienced by approximately 60%-90% of patients with psoriasis, such as itching, skin pain, and discomfort (6, 7). Indeed, excessive TRP channel activation by repeated capsaicin cream application results in the degeneration of the affected sensory nerve fibers, thus minimizing both psoriasis-associated skin lesions and cutaneous discomfort (8).

Imiquimod (IMQ)-induced psoriasiform skin inflammation is the most widely used animal model for studying the pathological mechanism of psoriasis. The exposure of the mouse skin to the Toll-like receptor 7 (TLR7) and TLR8 agonist IMQ can mediate phenotypic changes closely resembling those in psoriasis *via* the IL-23/IL-17 immune response (9). Moreover, this mouse model exhibits spontaneous scratching (10) and stress-induced exacerbation of lesions (11). It has recently been shown that sensory neurons regulate IMQ-induced psoriatic immune responses through ion channels, of which TRPV1 plays a pro-inflammatory role (12) and TRPA1 acts in a protective manner (13). However, these studies simply demonstrated the regulatory role of TRP channels on the integrated immune response through gene-knockout (KO) or selective antagonist application during psoriasis development but failed to define the “bridge” between inflammatory cells and nociceptive neurons in psoriasis.

A small-scale clinical trial showed that the inhibition of neuropeptide vesicle release by onabotulinumtoxinA was associated with alleviation of psoriatic lesions (14). In addition, sensory neuron-derived neuropeptides have been shown to mediate Type17 immune responses against fungal infection (15–17). Previously, we reviewed elevated innervation levels in patients with psoriasis (18). Based on these studies, we speculate that neuropeptides are involved in the interaction between sensory neurons and the Type17 immune response in psoriasis.

Here, we report that resiniferatoxin (RTX)-induced sensory denervation suppressed the Type17 immune response in an IMQ-induced psoriasiform dermatitis mouse model by decreasing initial IL-23 expression *via* nearly abolished neuron-derived CGRP. RTX is an ultrapotent TRPV1 agonist that causes specific ablation of nociceptive fibers. In this study, we demonstrate that pretreatment of mice with RTX significantly reduced IMQ-induced skin inflammation and nociceptive sensation. The elevated expression of neuron-derived CGRP induced by IMQ treatment was also significantly inhibited by sensory denervation. Most importantly, we confirm that RTX denervation-induced IL-23 expression downregulation was significantly reversed following introduction of CGRP.

Thus, we demonstrate that sensory neurons can regulate IL-23 expression in the pathology of psoriasis through neuron-derived CGRP, suggesting the relevance of alternative therapeutic strategies to target neuronal mechanisms and effectively control psoriasis.

## Materials and Methods

### Mice and Treatments

Mice (male. Balb/c) were purchased from the Animal Department of Capital Medical University and kept in the animal facility of Beijing Chaoyang Hospital under specific pathogen-free conditions, and provided with food and water ad libitum. All animal experiments were approved by the Ethics Committee of Beijing Chaoyang Hospital and Capital Medical University and all procedures were in accordance with the animal care guidelines of Capital Medical University.

Psoriasiform dermatitis was induced using Aldara (a pharmaceutical preparation of cream containing 5% IMQ; 3M Pharmaceuticals). The dorsal skin of mice at 8 to 10 weeks of age was shaved and depilated >3 days prior to the application. Mice received a daily topical dose of 62.5 mg Aldara on the back skin or the ear for 5 consecutive days. Control mice were treated similarly with a control vehicle cream (Vaseline). The first day of IMQ application was counted as Day 0. For neuropeptide inhibitor and agonist experimental *in vivo*, 10 ug of selective CGRP antagonist (CGRP_8-37_) (Bachem, Bubendorf, Switzerland) or 0.5ug CGRP (Bachem, Bubendorf, Switzerland) in 200 ul PBS was injected intradermally at spread 9 sites in dorsal skin at Day -2, Day -1 and then 2 hours prior to the IMQ treatment. PBS as a vehicle was given at the same way. For injections of recombinant mouse IL-23 (rIL-23), anaesthetized mice had intradermal injections of 500 ng rIL-23 (eBiosciences, CA, USA) in 25ul PBS or 25ul PBS as a vehicle was given into the dorsal sides of the ears on days 0, 2 and 4. For the intradermal injections, mice were initially anesthetized with 4% isoflurane until they stopped making a movement, then mice were continuously maintained with 1% isoflurane. Mice were euthanized and tissues were harvested for further analyses at the respective time points.

### Skin Inflammation Severity Scoring

For the back skin, the severity of skin inflammation was assessed every day with a scoring system Psoriasis Severity Index (PSI) Score considering erythema, scaling, and skin thickness. Each parameter was evaluated and scored with 0 to 4 (0: absent to 4: high severity). A cumulative score was generated from these parameters. Additionally, ear thickness was measured using a micrometer with 0.1 mm accuracy. The indicators were assessed at the respective time points.

### Histology

Skin tissue samples were formalin-fixed (10%) and embedded in paraffin. 4μm sections were stained with Hematoxylin and Eosin (H&E) using standard protocols. Histopathologic scoring assessing characteristic parameters in psoriasis was described previously (19). Briefly, scoring parameters and values were determined on the basis of the signs characteristic to psoriasis at 200x magnification: 1) Thickness of skin epidermal layer; 2) Number of cell rows in stratum granulosum; 3) Number of Munro’s microabscesses throughout the section. Average scores for these parameters were calculated to determine the composite histological score of tissue sections.

### Peripheral Sensory Denervation

To investigate the role of TRPV1-positive sensory fibers in the development of psoriasis-like dermatitis in mice, RTX (Cayman Chemical, Michigan, USA) was injected subcutaneously into the flank of mice at 4-5 weeks of age in at 30, 70 and 100 mg/kg dose on 3 consecutive days. Mice were then allowed to rest for 4 weeks before the IMQ treatment. The success of RTX denervation was confirmed by the lack of wiping movements in response to 20ul of 0.1% capsaicin administration into the eye.

### Behavioral Assessment

Mice were briefly moved to the observe chamber for at least 60 min prior to every behavior recording for acclimatization and baseline behaviors were assessed 1 day before IMQ or Vaseline application. During treatment period, mice behaviors were videotaped for 30 min to 40 min using camera, at 4h after each topical application. The videotapes were played back and the number of nociceptive behaviors in 20 min was counted by a trained investigator blinded to the treatment. Nociceptive behavior was defined as below: a scratch was counted when a mouse lifted its hind paw to scratch the treated dorsal region and returned the paw to the floor or to the mouth; a biting/licking was counted as touching of the mouth and nose to the treated dorsal area; a flinching was defined as a rotating/rippling movement. Total spontaneous behaviors were calculated as the total number of nociceptive behaviors indicating psoriasis-associated cutaneous discomfort in this model.

### CGRP Release Assay From Skin Explants

A skin punch biopsy (12 mm) was collected from the dorsal skin samples of the euthanized mice, and quickly transferred to 24-well plates containing 1 mL of DMEM (Gibco). Incubated the explants at 32°C with gentle shaking (150 rpm) for 30 minutes. Then, the bath supernatant from the organ cultures was collected, and the CGRP EIA kit (Cayman Chemical, Michigan, USA) was used to perform an assay to determine the CGRP concentration according to the manufacturer’s instructions.

### Flow Cytometry

For single-cell suspensions of dorsal skin, tissue samples were minced and incubated for 2 h (37°C, shaking) in DMEM (Gibco) supplemented with Brefeldin A (BD biosciences, California, USA), 2% FCS, 100 mg ml Liberase TM (Roche), 100mg/ml DNase I (Roche) and 0.5mg/ml Hyaluronidase (Sigma-Aldrich, Missouri, USA). After enzymatic digestion, the mixture was filtered through a 70μm cell strainer (BD), which then resuspended in FACS buffer (PBS with 2 mM EDTA and 2% FCS) for analysis. Single-cell suspensions were pre-incubated with Fc Block (clone 2.4G2), and then incubated with the following antibodies: anti-CD90.2 (Thy-1.2)-eFluor 450 (eBioscience, California, USA), anti-TCR gamma/delta-APC (eBioscience, California, USA), anti-TCR beta-PE (eBioscience, California, USA), anti-CD45.2-PerCP/Cy5.5 (eBioscience, California, USA). Cells were then fixed and permeabilized using BD Cytofix/Cytoperm kit (BD biosciences, California, USA) as per the manufacturer’s instructions. Cells were stained in Perm/Wash buffer with anti-IL-17A-Alexa Fluor 488 (eBioscience, California, USA). Samples were analyzed on BD FACSCanto™ II Flow Cytometry System (BD, New Jersey, USA). Data were analyzed using FlowJo software (TreeStar, Oregon, USA).

### Protein and RNA Isolation & Quantitation

Dorsal skin and dorsal root ganglia (T3-12) samples were collected at various time points. For measurement of cytokine protein, skin tissue was homogenized in 1mL Cell extraction buffer (Invitrogen, California, USA) supplemented with phenylmethylsulfonyl fluoride (Roche, Basel, Switzerland) and protease inhibitor (Sigma-Aldrich, Missouri, USA) according to the manufacturer’s instructions. Tissue homogenates were centrifuged for 10 minutes at 7500 rpm. Measurement of the levels of inflammatory cytokines was completed using ELISA according to the suggested protocol of the manufacturer: IL-23, IL-6, IL-17A (ELISA MAX Set; Biolegend, California, USA). For PCR, mRNA was isolated using the RNAsimple Total RNA Kit (Tiangen, Sichuan, China) following the manufacturer’s protocol. First strand cDNA synthesis was achieved using All-in-one 1^st^ Strand cDNA Synthesis Kit (Novoprotein, Shanghai, China). Realtime qRT-PCR was performed using SYBR qPCR SuperMix Plus Kit (Novoprotein, Shanghai, China), supplemented with gene specific primers, quantitative RT-PCR was performed with ABI 7500 real-time PCR instrumentation (Life Technologies). All samples were analyzed at least in duplicate, and CT values are normalized to β-actin expression and are shown as relative expression (2^-△Ct^). Primers used include: *β-actin* (Fwd: 5’-GTGACGTTGACATCCGTAAAGA-3’; Rev:5’-GTAACAGTCCGCCTAGAAGCAC-3’); *IL-23* (Fwd: 5’-CCAGCGGGACATATGAATCTACT-3’; Rev:5’-TGTCCTTGAGTCCTTGTGGG-3’); *IL-17A* (Fwd: 5’-TCCAGAAGGCCCTCAGACTA-3’; Rev:5’-AGCATCTTCTCGACCCTGA-3’); *IL-6* (Fwd: 5’-CTGCAAGAGACTTCCATCCAG-3’; Rev:5’-AGTGGTATAGACAGGTATGTTGG-3’); *Substance P* (Fwd: 5’-GCAGAGGAAATCGATGCCAAC-3’; Rev:5’-CCATGTCCAGCATCCCGCTT-3’); *CGRP* (Fwd: 5’-CCAGTGGGTGAGGAGAAAGTC-3’; Rev:5’-AAGCAAGACTAGAAGCTCTACTAGG-3’); *TRPV1* (Fwd: 5’-CGACACCATTGCTCTGCTCCTG-3’; Rev:5’- GGTCACCAGTGCCATGTTCCG-3’); *TRPA1* (Fwd: 5’-AGGTGATTTTTAAAACATTGCTGAG -3’; Rev:5’- CTCGATAATTGATGTCTCCTAGCAT-3’).

### Statistical Analysis

Statistical analysis were performed with SPSS 19.0 (SPSS Inc., Chicago, USA). Graphs were created using GraphPad Prism (GraphPad Software, California, USA) software. Data are represented as means ± standard deviations. Differences between groups were compared using Student’s t-test or two-way ANOVA with Bonferroni *post hoc* test as indicated. *P<0.05* accepted as significant.

## Results

### RTX-Mediated Sensory Denervation Reduces IMQ-Induced Psoriasiform Dermatitis and Nociceptive Behavior

**Figure 1:**
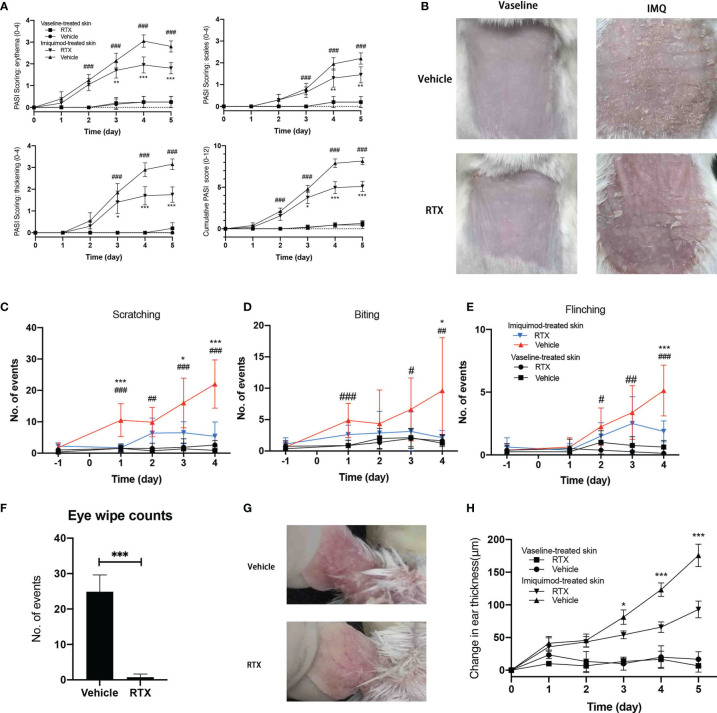
RTX-mediated sensory denervation protects mice from IMQ-induced psoriasiform dermatitis and nociceptive behavior. **(A)** Skin erythema, scaling, and thickness score were daily assessed on a scale from 0 to 4, and the cumulative score was depicted (n = 9 mice per time point). **(B)** Representative images of the back skin of vaseline- and IMQ-treated innervated mice *vs*. denervated mice on day 5 are shown. **(C–E)** The number of scratch, licking/biting, and flinching behavior in 30 min was daily counted and presented at indicated time points (n = 9 mice per time point). **(F)** The number of eye wiping movements in response to capsaicin in 2 min (n = 9). **(G)** Representative images of the ear skin of IMQ-treated innervated mice *vs*. denervated mice on day 5 are shown. **(H)** Ear thickness was measured at indicated time points and presented the increase in the thickness compared with day 0 (n = 8 mice per time point). Significance was calculated using a two-way ANOVA for (a. c-e, h). RTX+IMQ *vs* vehicle+IMQ *P < 0.05; **P < 0.01; ***P < 0.001. vehicle+IMQ *vs* vehicle+vaseline ^#^P < 0.05; ^##^P < 0.01; ^###^P < 0.001. Significance was calculated using unpaired Student’s t test for **(F)** vehicle *vs* RTX, ***P < 0.001.

Repeated topical IMQ application to the shaved dorsal skin of mice resulted in cutaneous inflammation characterized with erythema, scaling, and thickening (**Figure 1A**), consistent with the observations reported in clinical psoriasis (**Figure 1B**). Nociceptive behavior, identified as licking (**Figure 1C**), biting(**Figure 1D**), or flinching (**Figure 1E**) of the treated dorsal area, was more pronounced after IMQ treatment than that after vaseline treatment and gradually increased with the development of psoriasis-like dermatitis.

Nociceptive sensations are transmitted by small-fiber sensory neurons (20). To investigate the contribution of sensory neurons in this model, mice were systemically pre-treated with RTX to induce small-fiber sensory neuropathy (21). RTX can specifically deplete TRPV1-positive neurons and their corresponding nerve terminals in the skin (22) without affecting their expression in skin non-neuronal resident cells (**Supplementary Figure 1**). Therefore, RTX treatment seemed to specifically ablate TRPV1-positive nociceptors. The success of sensory denervation was confirmed from the lack of eye wiping movements in response to application of eye drops containing 0.1% capsaicin (**Figure 1F**) before IMQ treatment.

Sensory denervation significantly reduced the development of psoriasis-like lesions, as evident from decreased erythema and skin thickening (**Figure 1A**) from day 3 to day 5 and decreased scaling from day 4 to day 5. Consistent with the observed improvement in psoriasis-like dermatitis, sensory denervation significantly reduced the nociceptive behavior in mice (**Figures 1C–E**). To eliminate the possibility of the influence of reduced nociceptive behavior, we applied IMQ to the ear skin that is difficult to scratch or lick. As expected, RTX denervation exhibited similar protective effects on IMQ-induced psoriasis-like dermatitis in the ear skin (**Figures 1G, H**). Together, these results suggest that sensory neuron depletion inhibits the progression of psoriasiform dermatitis and protects mice from IMQ-induced nociceptive behavior.

### Histologic Analysis Confirms the Protective Effect of Sensory Denervation in IMQ-Induced Psoriasiform Dermatitis

**Figure 2:**
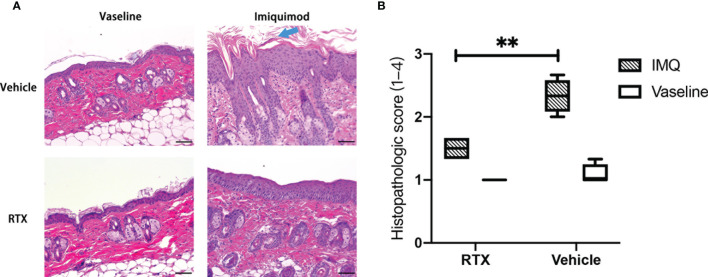
Representative histologic view of denervated and innervated mouse dorsal skin tissues after IMQ treatment. **(A)** Representative histopathology images of the skin of vaseline- and IMQ-treated innervated mice *vs*. denervated mice on day 5 at ×200 magnification are shown (Scale bar, 100 μm). **(B)** Histopathologic score of vaseline- or IMQ-treated dorsal skin samples from denervated or innervated mice (n = 5). Significance was calculated using a two-way ANOVA, RTX+IMQ *vs* vehicle+IMQ, **P < 0.01.

The histopathological changes in IMQ-induced skin lesions were evaluated using hematoxylin and eosin (H&E) staining. IMQ-treated mouse skin showed characteristics of human psoriasis(**Figure 2A**), including hyperproliferation of keratinocytes, hyperkeratosis, parakeratosis, and Munro’s microabscesses (**Figure 2A**, blue arrow). These results indicate that the denervation induced by RTX could reduce the infiltration of inflammatory cells in psoriatic lesions. In addition, histopathologic scoring of the characteristic hallmarks of psoriasis significantly decreased in IMQ-treated skin samples from RTX-induced denervated mice (**Figure 2B**).

### RTX Denervation Suppresses Type17 Immune Response by Downregulating IL-23 Expression in IMQ-Treated Skin

**Figure 3:**
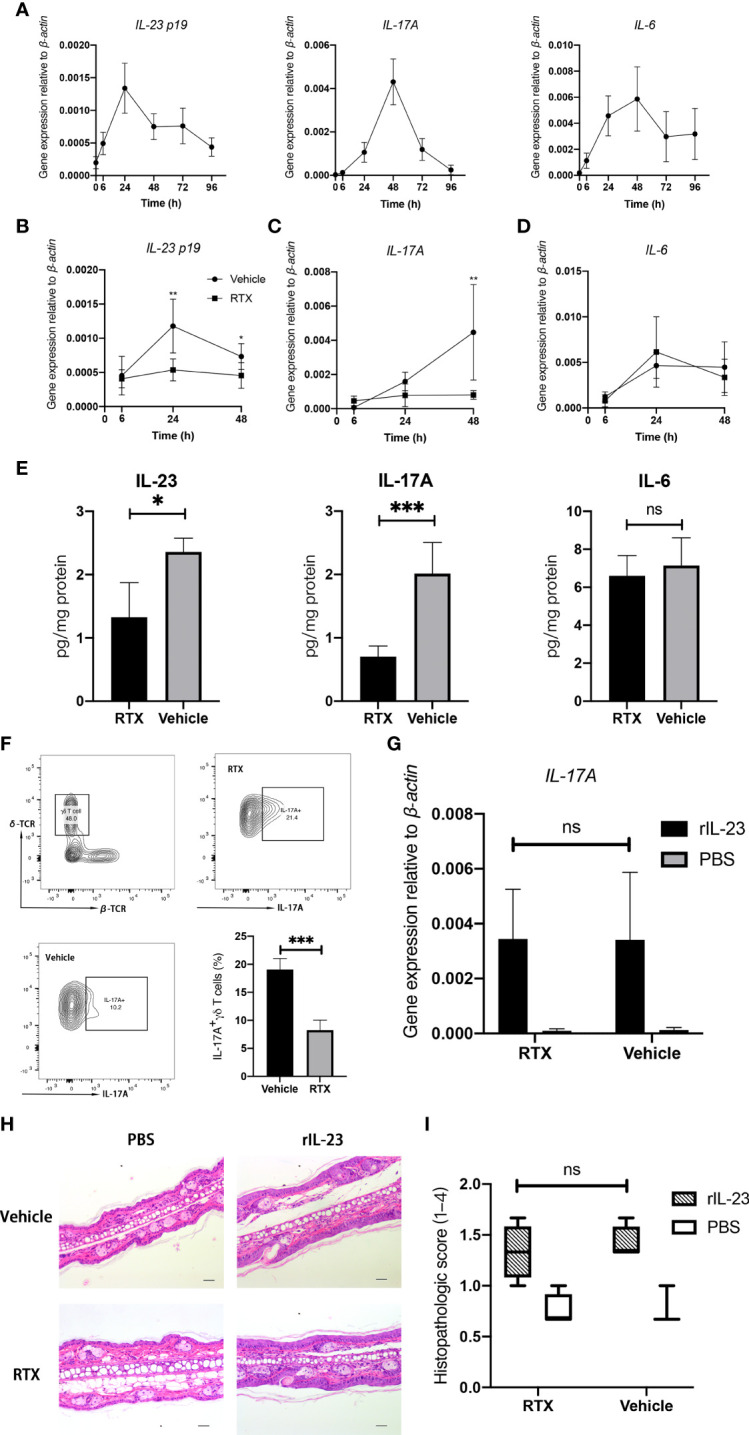
RTX denervation suppresses Type17 immune response in the IMQ-treated skin. **(A)** IMQ transiently induces the mRNA expression of *IL-23*, *IL-6*, and *IL-17A* in the skin during IMQ treatment period (n = 5). **(B–D)** The mRNA expression of *IL-23*, *IL-6*, and *IL-17A* in the skin from RTX + IMQ or vehicle + IMQ-treated mice at the indicated time points (n = 9). **(E)** The protein levels of IL-23, IL-6, and IL-17A in the whole dorsal skin from RTX +IMQ - or vehicle + IMQ -treated mice were measured at day 3 (n = 8). **(F)** The percentage of IL-17A-expressing γδ T cells was determined in the whole dorsal skin from RTX- or vehicle-treated mice on day 4 (n = 5). **(G)** Mice were pre-treated with RTX or vehicle and then challenged with recombinant IL-23 or PBS at D0, D2, and D4. After 24 h from the last injection, the skin was harvested from injection sites to measure *IL-17A* mRNA expression (n = 6). **(H)** Representative histopathology images of the skin of PBS- and rIL-23-treated innervated mice *vs*. denervated mice on day 6 at ×200 magnification are shown (Scale bar, 100 μm). **(I)**. Histopathologic score of PBS- and rIL-23-treated dorsal skin samples from denervated or innervated mice (n = 4). Significance was calculated using a two-way ANOVA for **(B–D)** RTX+IMQ *vs* vehicle+IMQ, and for **(G, I)** RTX+rIL-23 *vs* vehicle+rIL-23. Significance was calculated using unpaired Student’s t test **(E, F)**, RTX- versus vehicle-treated groups. *P < 0.05; **P < 0.01; ***P < 0.001; NS, not significant.

IMQ-induced cutaneous inflammation is thought to be related to Type17 immune response (23). We confirmed the dynamic changes in proinflammatory cytokine expression during IMQ treatment (**Figure 3A**). The mRNA expression of *IL-23* peaked at 24 h, whereas that of *IL-6* and *IL-17A* peaked at 48 h. After 96 h, the mRNA expression levels of all cytokines started to decline to baseline levels.

To identify the potential mechanisms underlying the improvement in psoriasiform dermatitis following denervation, we examined changes in the expression of inflammatory cytokines in IMQ-induced mice after RTX denervation. The mRNA expression of *IL-23* and *IL-17A* was significantly downregulated in RTX-treated mice as compared with that in vehicle-treated mice, reaching maximal differences at 24 h (**Figure 3B**) and 48 h (**Figure 3C**), respectively. However, no statistically significant change in *IL-6* mRNA expression was observed (**Figure 3D**). The protein expression levels of IL-23, IL-6, and IL-17A in the digested dorsal skin were consistent with the mRNA expression result (**Figure 3E**). Hence, the trends in cytokine expression reflect the alleviation in the skin pathology in sensory denervated mice, initiated as early as during the second IMQ application.

Compared with alpha:beta T cells (including CD4^+^ and CD8^+^ T cells), gama:deta T cells has a higher portion (47.7% *vs* 17.6%) in CD45^+^ Thy1^+^ T-cell and also more IL-17A-possitive (19.2% *vs* 0.43%)(**Supplementary Figure 2**). This phenomenon confirmed that, IL-23-responsive dermal γδ T cells are the major IL-17 producers in the mouse skin (24). We investigated if there was any decrease in the number of IL-17-producing γδ T cells in IMQ-treated mice after sensory denervation by evaluating the prevalence of IL-17A-positive γδ T cells by flow cytometry. A significant decrease in the proportion of IL-17A-positive γδ T cells was observed in the dorsal skin tissue after RTX denervation (**Figure 3F**). Considering that the expression of IL-17 is highly dependent on IL-23, we administered recombinant mouse IL-23 to the ear dorsal skin of RTX- and vehicle-treated mice to identify whether sensory denervation exerts any direct effect on IL-17-producing cells. However, RTX denervation failed to influence *IL-17A* mRNA expression induced by recombinant IL-23 (**Figure 3G**). Simultaneously, intradermal IL-23 injections triggered the development of psoriasiform skin, which was unaffected by RTX-induced denervation (**Figures 3H, I**). These results suggest that the depletion of sensory neurons in the skin may significantly suppress the typical immune response in psoriasis-like dermatitis by reducing the generation of IL-23.

### Sensory Denervation Results in the Loss of CGRP Expression in IMQ-Induced Psoriasis-Like Dermatitis Model

**Figure 4:**
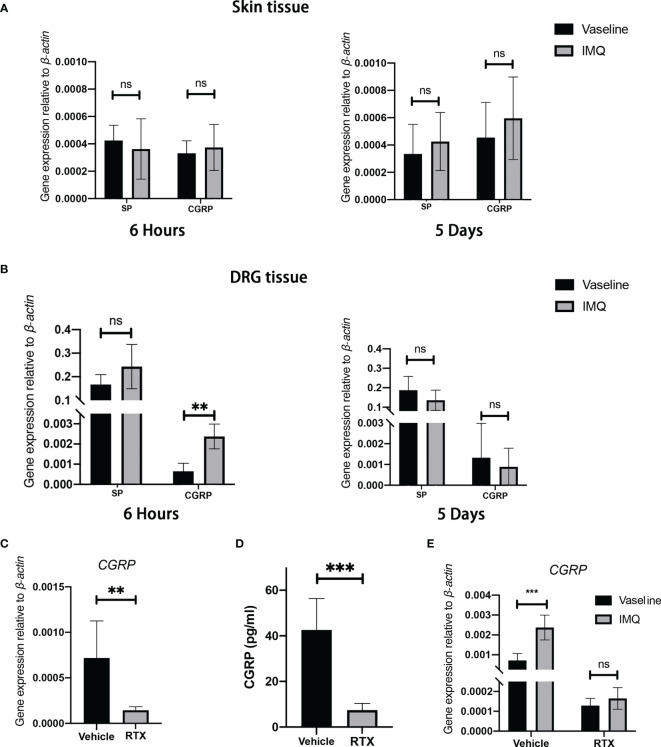
Sensory denervation influences the level of CGRP in an IMQ-induced psoriasis-like dermatitis model. **(A, B)** The mRNA expression of *SP* and *CGRP* in the whole back skin **(A)** and DRG **(B)** after 5 days or 6 hours from IMQ application was measured (n = 8-9). **(C)** Decreased levels of *CGRP* mRNAs in the DRGs after RTX treatment as compared with those in control animals (n = 9). **(D)** Decreased CGRP protein levels in dorsal skin tissue after RTX treatment as compared with those in control animals (n = 6). **(E)** IMQ-induced transient elevation in *CGRP* mRNA expression (6 hours) was inhibited by RTX treatment (n = 6). Significance was calculated using unpaired Student’s t test for **(A, B, E)** IMQ *vs* vaseline and **(C, D)** RTX *vs* vehicle. **P < 0.01; ***P < 0.001, NS, not significant.

Studies have reported elevated levels of neuropeptides (CGRP and substance P) in psoriatic lesions (25, 26). We measured the mRNA expression of neuropeptides in the treated dorsal skin and dorsal root ganglion (DRG) located at anatomical sites T3-12. Unexpectedly, no differences in the expression of substance P or CGRP were observed between 5-day IMQ treatment and vehicle treatment groups (**Figure 4A**). Considering that IMQ-induced cutaneous inflammation is evident after every 24 h of IMQ application during the entire 5-day experimental period, we focused on the dynamic neuropeptide expression at the early stage of IMQ application. We found that the expression of CGRP (3.72-fold, P < 0.0001; **Figure 4B**) significantly increased in the DRG of IMQ-induced mice at 6 h following the initiation of topical application; however, no statistically significant changes in substance P expression were observed in the skin or DRG tissue (**Figure 4B**). Nerve-derived neuropeptides are synthesized in the neuronal cell body and are anterogradely transported to the skin where they are secreted. To examine the dynamic changes in neuron-derived CGRP in this model, we used a modified *ex vivo* skin organ culture method (27). Unexpectedly, in contrast to the observed changes in mRNA expression, we noted a gradual decrease in the level of neuron-derived CGRP in the skin tissue during the entire IMQ treatment phase (**Supplementary Figure 3**). Hence, these results suggest an early and transient increase in CGRP expression in this model.

To determine the effect of sensory denervation on the levels of CGRP in this model, we examined the changes in CGRP expression after RTX denervation. As expected, the mRNA expression of *CGRP* in the DRG tissue significantly decreased (**Figure 4C**) and the secretion of CGRP from nerve fibers was nearly completely abolished in RTX-treated mice as compared with that in vehicle-treated mice (**Figure 4D**). In addition, RTX inhibited IMQ-induced transit elevation in *CGRP* mRNA expression in the DRG (**Figure 4E**). From these data, it is clear that RTX-induced sensory denervation resulted in a significant decrease in the level of CGRP in the skin.

### TRPV1-Positive Neurons Regulate the Expression of IL-23 in IMQ-Induced Model *via* CGRP

**Figure 5:**
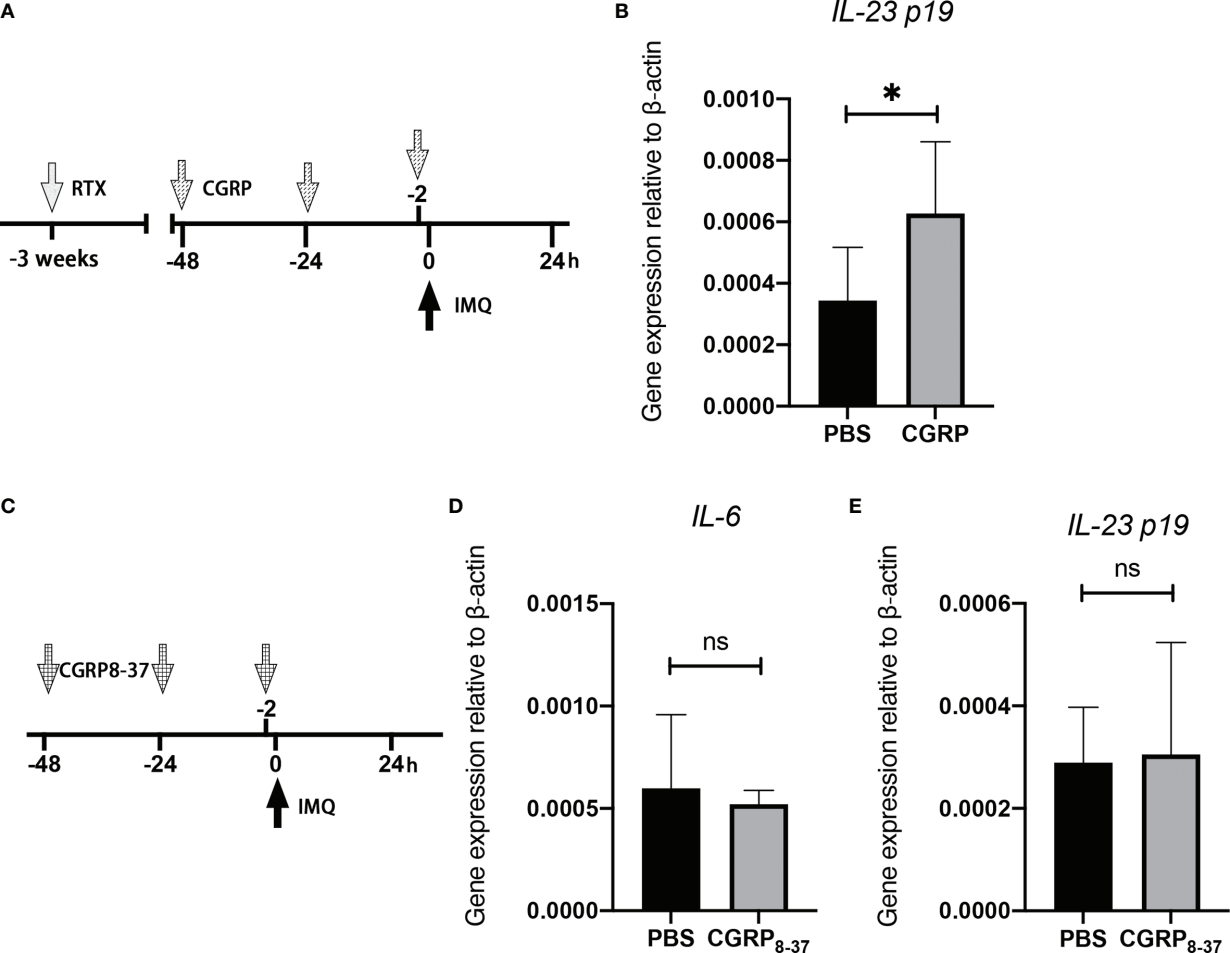
CGRP receptor agonist administration into the denervated skin reverses the decreased expression of IL-23. **(A)** Representation of the experimental schematic indicating the daily injection of CGRP, or PBS before topical applications of IMQ on RTX-treated mice. **(B)** The mRNA expression of *IL-23* was evaluated in PBS- or CGRP-treated denervated mice at day 1 (n = 8-9). **(C)** Representation of the experimental schematic indicating the daily injection of CGRP_8-37_ or PBS before topical applications of IMQ. **(D, E)** The mRNA expression of *IL-6* and *IL-23* was evaluated in PBS- or CGRP_8-37_-treated mice on day 1 (n = 8-9). Significance was calculated using unpaired Student’s t test, RTX *vs* vehicle, *P < 0.05, NS, not significant.

To determine the potential contribution of CGRP to the denervation-mediated decrease in IL-23 expression in the IMQ-treated skin, CGRP or phosphate-buffered saline (PBS) was intradermally injected into the dorsal skin region after RTX denervation (**Figure 5A**). The choice of doses and administration routes was based on previous reports, which showed the effectiveness of mediating skin biological reactions *in vivo* (28). We found that the intradermal administration of CGRP in RTX-treated mice restored IL-23 expression at day 1 (**Figure 5B**), as expected. However, injection of CGRP_8-37_ alone into the innervated dorsal skin (**Figure 5C**) failed to suppress IMQ-induced IL-23 and IL-6 expression at day 1 (**Figures 5D, E**). Together, these data show that TRPV1-positive neurons derived from CGRP played a significant conditional role in the expression of IL-23 in the IMQ-induced model.

## Discussion

In the current study, we provide the evidence of the modulation of IL-23 expression by TRPV1-positive sensory neurons *via* CGRP that affects their central role in the onset of IMQ-induced psoriasiform dermatitis. In the development of IMQ-induced psoriasiform dermatitis, intact TRPV1-positive neurons are essential for the initial Type17-mediated inflammation. Ablation of nociceptors reduced the proportion of IL-17-producing γδ T cells through CGRP-dependent regulation of IL-23 expression. Thus, we have defined the TRPV1^+^ sensory neurons/CGRP/IL-23-producing cells/γδ T cell axis that provides a critical neuron-immune response at the early stage of IMQ-induced psoriasis inflammation.

We demonstrate the dynamic changes in the expression of IL-23, IL-6, IL-17, and γδ T17 during the IMQ treatment phase. Mice receiving IMQ exhibited a significant increase in itch-associated behavior. We have previously reviewed that elevated pro-inflammatory factors in psoriasis lesion could cause unfaithful activation of nociceptive neurons, resulting in annoying sensations such as pain, pruritus, and hyperalgesia (18). This may account for the gradual increase in nociceptive behavior along with the development of psoriasis-like dermatitis during IMQ treatment.

We used systemic RTX treatment as an established technique to specifically ablate capsaicin-sensitive primary afferent neurons, without affecting non-neural cells, by permanently opening the non-selective cation channel (TRPV1, TRPA1), resulting in the loss of further responsiveness of these neurons (29, 30). We confirmed that RTX-mediated denervation protected against IMQ-induced skin pathology and cutaneous discomfort. This is consistent with the clinical phenomenon of spontaneous improvement in psoriatic skin lesions after central or peripheral nerve damage (4).

IMQ-induced psoriasis-like cutaneous inflammation is a widely used psoriasis model. To the best of our knowledge, this is the first study to show dynamic changes in CGRP level in this model. CGRP, a member of the calcitonin family of peptides, is expressed in both peripheral and central neurons and deeply involved in the development of psoriasis. Several studies have demonstrated elevated CGRP expression in psoriatic lesions (25, 26, 31). In addition, psoriasis patients with high level of psychological stress exhibit elevated CGRP-immunoreactive nerves in the papillary dermis of lesional skin (32). Further, psoriasis patients with pruritus also showed higher expression of CGRP receptors than non-pruritic patients (33). Neuropeptides (34) and various TLRs (35–39) are co-expressed in TRPV1-positive neurons. In addition, nociceptive neurons can sense the environment through TRPV1 and TLRs (40, 41). IMQ can induce inward currents in DRG neurons in a concentration-dependent manner, thereby elevating their excitability and maintaining their hypersensitive state *in vitro* (39). Together, these studies may explain the instantaneously elevated mRNA expression of *CGRP* in the DRG after 6 h from the initiation of topical IMQ application. TLR signaling can prompt the secretion of neuropeptides by sensitizing TRPV1 channels (42). However, we found decreased neuron-derived CGRP during the 5-day IMQ treatment. This may be associated with the exhausted vesicle release of CGRP, as the density of CGRP-positive peptidergic fibers significantly decreased after IMQ treatment (43). This reflects that the IMQ model mimics acute psoriasis, as the number of peptidergic nerve fibers in the skin lesions significantly decreased during the exacerbation phase of psoriasis (44). It can’t be denied that keratinocyte is also a source of CGRP in the skin tissue (45). However, compared with the DRG tissue, the mRNA expression of CGRP in the skin tissue is low (nearly 10%) and is also unaffected by IMQ treatment. Considering that RTX denervation nearly abolishes the content of CGRP released from dorsal skin explants, we speculate that, instead of keratinocyte-derived CGRP, CGRP secreted by sensory neuron fibers may regulate the initial immune response at the early stage of IMQ-induced dermatosis.

CGRP has been reported to play an essential role in the induction of Type17 immune response. Macrophages, mast cells, dendritic cells, and γδ T cells express the functional CGRP receptor, calcitonin receptor-like receptor (CRLR), and its receptor activity-modifying protein 1 (RAMP1) (46). Here, we found that IMQ-induced elevated mRNA expression of *IL-23* was significantly inhibited by RTX denervation. Moreover, intradermal injections of CGRP peptide into the denervated skin reversed the decreased expression of IL-23. Therefore, we speculate that neuron-derived CGRP is essential for the initial expression of IL-23 in this model. We also show that sensory denervation significantly decreased the mRNA and protein expression of IL-17A and the proportion of IL-17A-positive γδ T cells, the major cellular source of IL-17A in this model (24). By binding to RAMP1, CGRP directly upregulates IL-17 production (47). While, we found that RTX denervation failed to influence the rIL-23-induced expression of IL-17A, indicating that sensory neurons failed to directly regulate the function of γδ T cells. CGRP is thought to stimulate endothelial cells to produce IL-6, which polarizes the outcome of antigen presentation by Langerhans cells to CD4^+^ T cells toward a Th17 response *in vivo* (48). Consistent with previous studies (49), we also found that IL-6, the expression of which is intact after sensory denervation, is dispensable for both the development and activation as well as recruitment of IL-17-possitive γδ T cells in this mouse model. Based on these findings, we infer that sensory neuron-derived CGRP participates in the onset of Type17 immune response through the regulation of IL-23 expression in psoriasis development. We also explored whether long-term intradermal injection of CGRP can restore the denervation-induced alleviation on psoriatic lesion, while repeatedly application of IMQ on skin result in the infiltration of inflammatory cell and the proliferation of blood vessels in the superficial dermis. These histological changes make the operation of long-term intradermal injection of CGRP receptor antagonist or CGRP difficult and traumatic. It’s unexpected that, administration of CGRP_8-37_, a well-characterized selective receptor antagonist, into the innervated skin failed to mimic the denervation-mediated downregulation in IL-23 expression after IMQ treatment. *In vitro*, we confirmed that CGRP have direct immunomodulatory effect on dendritic cells (prompt the expression of IL-23), which can be abolished by CGRP_8-37_ (submitted and under review). In addition, the potent effect of CGRP_8-37_ as a selective CGRP antagonist was also confirmed by previous studies (27, 28, 50). Hence, we speculate that blocking a single type of neuropeptide through selective receptor antagonists may be insufficient against various neuropeptides secreted by neurons (**Supplementary Figure 4**) in the initial phase of IMQ-induced IL-23 expression, owing to the conflicting immune microenvironment.

## Conclusion

These results show that the nociceptive sensory neuron-derived neuropeptide CGRP is essential in the initial stage of the Type17 immune response in IMQ-induced psoriasis-like dermatitis. We provide evidence of the interplay between sensory neurons, CGRP, and IL-23-producing cells in the development of psoriasis. Botulinum neurotoxin A (BoNT/A), which prevents vesicle fusion and release of neuropeptides from neurons, reduces CGRP level (51) and exhibits beneficial clinical effects (14). However, further studies are warranted to explore the involvement of this complex interplay of neurons and immune cells in psoriasis to design potential therapeutic interventions for psoriasis.

## Data Availability Statement

The raw data supporting the conclusions of this article will be made available by the authors, without undue reservation.

## Ethics Statement

The animal study was reviewed and approved by Ethics Committee of Beijing Chaoyang Hospital and Capital Medical University.

## Author Contributions

YH designed the study, critically revised the paper, and guarantee the study’s integrity. XZ and JC contributed equally to this study, conducted most of the experiments, analyzed the data, and wrote the manuscript. SZ and XY helped write the manuscript. JD and SZ analyzed the data. YT and TY supervised the study. All authors contributed to the article and approved the submitted version.

## Funding

This research was funded by National Natural Science Foundation of China (81773314), and by Beijing Natural Science Foundation (7172082).

## Conflict of Interest

The authors declare that the research was conducted in the absence of any commercial or financial relationships that could be construed as a potential conflict of interest.

## Publisher’s Note

All claims expressed in this article are solely those of the authors and do not necessarily represent those of their affiliated organizations, or those of the publisher, the editors and the reviewers. Any product that may be evaluated in this article, or claim that may be made by its manufacturer, is not guaranteed or endorsed by the publisher.

## Supplementary Material

Supplementary Figure 1 The mRNA expression of *TRPV1* and *TRPA1* in the DRG and dorsal skin tissue (n = 9). RTX can significantly decreased the expression of *TRPV1* and *TRPA1* in the DRG tissue without affecting their expression in skin non-neuronal resident cells.

Supplementary Figure 2 The percentage of IL-17A-expressing cells from IMQ-treated dorsal skin tissue at Day4. (Representative FACS plot from five mice analyzed).

Supplementary Figure 3 CGRP protein levels in the *ex vivo* dorsal skin culture supernatant during IMQ treatment period at the indicated time points (n = 6).

Supplementary Figure 4 RTX-denervation significantly decreased the mRNA expression of neuropeptide SP and NPY in the DRG tissue (n=8-9).
