# Supplementary figures and images for: Nociceptive Sensory Fibers Drive Interleukin-23 Production in a Murine Model of Psoriasis via Calcitonin Gene-Related Peptide
Source: Front Immunol. 2021 Oct 22;12:743675. doi: 10.3389/fimmu.2021.743675 (PMC8569654; doi:10.3389/fimmu.2021.743675)

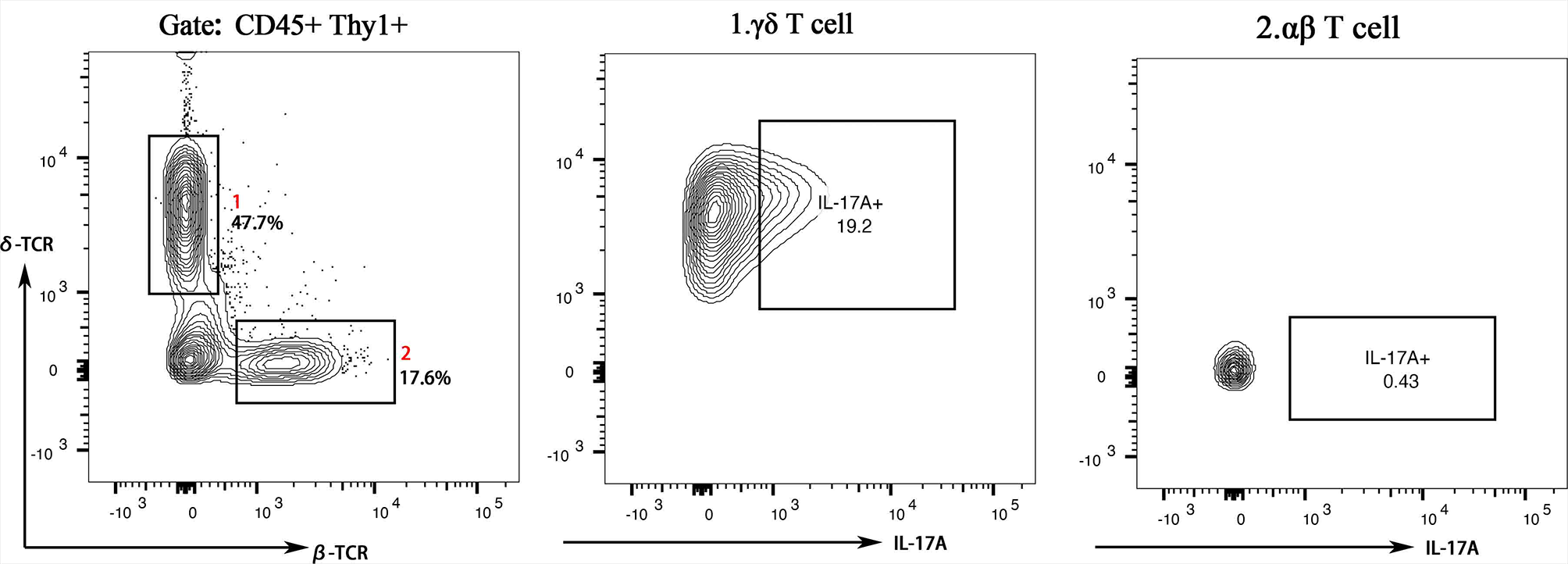

Supplement: Supplementary Figure 2 — The percentage of IL-17A-expressing cells from IMQ-treated dorsal skin tissue at Day4. (Representative FACS plot from five mice analyzed). [file Image_2.tif]
